# Detection Gap of Right-Asymmetric Neuronal Degeneration by CERAD Test Battery in Alzheimer’s Disease

**DOI:** 10.3389/fnagi.2021.611595

**Published:** 2021-02-02

**Authors:** Annika Kreuzer, Julia Sauerbeck, Maximilian Scheifele, Anna Stockbauer, Sonja Schönecker, Catharina Prix, Elisabeth Wlasich, Sandra V. Loosli, Philipp M. Kazmierczak, Marcus Unterrainer, Cihan Catak, Daniel Janowitz, Oliver Pogarell, Carla Palleis, Robert Perneczky, Nathalie L. Albert, Peter Bartenstein, Adrian Danek, Katharina Buerger, Johannes Levin, Andreas Zwergal, Axel Rominger, Matthias Brendel, Leonie Beyer

**Affiliations:** ^1^Department of Nuclear Medicine, University Hospital, Ludwig-Maximilians-University Munich, Munich, Germany; ^2^Department of Neurology, University Hospital, Ludwig-Maximilians-University, Munich, Germany; ^3^Department of Radiology, University Hospital, Ludwig-Maximilians-University, Munich, Germany; ^4^Institute for Stroke and Dementia Research, University Hospital, Ludwig-Maximilians-University, Munich, Germany; ^5^Department of Psychiatry, University Hospital, Ludwig-Maximilians-University, Munich, Germany; ^6^DZNE—German Center for Neurodegenerative Diseases, Munich, Germany; ^7^Munich Cluster for Systems Neurology (SyNergy), Munich, Germany; ^8^Ageing Epidemiology (AGE) Research Unit, School of Public Health, Imperial College, London, United Kingdom; ^9^Department of Nuclear Medicine Inselspital, University of Bern, Bern, Switzerland

**Keywords:** cognitive performance, Alzheimer’s disease, FDG-PET, hippocampal atrophy, asymmetry

## Abstract

**Objectives**: Asymmetric disease characteristics on neuroimaging are common in structural and functional imaging of neurodegenerative diseases, particularly in Alzheimer‘s disease (AD). However, a standardized clinical evaluation of asymmetric neuronal degeneration and its impact on clinical findings has only sporadically been investigated for F-18-fluorodeoxyglucose positron emission tomography (F-18-FDG-PET). This study aimed to evaluate the impact of lateralized neuronal degeneration on the detection of AD by detailed clinical testing. Furthermore, we compared associations between clinical evaluation and lateralized neuronal degeneration between FDG-PET hypometabolism and hippocampal atrophy. Finally, we investigated if specific subtests show associations with lateralized neuronal degeneration.

**Methods**: One-hundred and forty-six patients with a clinical diagnosis of AD (age 71 ± 8) were investigated by FDG-PET and the “Consortium to Establish a Registry for Alzheimer’s disease” (CERAD) test battery. For assessment of neuronal degeneration, FDG-PET hypometabolism in brain regions typically affected in AD were graded by visual (3D-surface projections) and semiquantitative analysis. Asymmetry of the hippocampus (left-right) in magnetic resonance tomography (MRI) was rated visually by the Scheltens scale. Measures of asymmetry were calculated to quantify lateralized neuronal degeneration and asymmetry scores were subsequently correlated with CERAD.

**Results**: Asymmetry with left-dominant neuronal degeneration to FDG-PET was an independent predictor of cognitive impairment (visual: *β* = −0.288, *p* < 0.001; semiquantitative: *β* = −0.451, *p* < 0.001) when controlled for age, gender, years of education and total burden of neuronal degeneration, whereas hippocampal asymmetry to MRI was not (*β* = −0.034; *p* = 0.731). Direct comparison of CERAD-PET associations in cases with right- and left-lateralized neuronal degeneration estimated a detection gap of 2.7 years for right-lateralized cases. Left-hemispheric neuronal degeneration was significantly associated with the total CERAD score and multiple subscores, whereas only MMSE (semiquantitative: *β* = 0.429, *p* < 0.001) and constructional praxis (semiquantitative: *β* = 0.292, *p* = 0.008) showed significant associations with right-hemispheric neuronal degeneration.

**Conclusions**: Asymmetry of deteriorated cerebral glucose metabolism has a significant impact on the coupling between neuronal degeneration and cognitive function. Right dominant neuronal degeneration shows a delayed detection by global CERAD testing and requires evaluation of specific subdomains of cognitive testing.

## Introduction

Alzheimer’s disease (AD) is the most common neurodegenerative disease, causing an enormous burden on patients, relatives, and caregivers, and the whole health care system (De Deyn et al., 2011; Colucci et al., 2014; Peña-Longobardo and Oliva-Moreno, 2015; Marečová and Zahálková, 2016). Accurate early diagnosis and prediction of further cognitive deterioration is essential. F-18-fluorodeoxyglucose positron emission tomography (F-18-FDG-PET) of the brain has been a decisive diagnostic tool for several years (Sperling et al., 2011; Perani et al., 2014; Salmon et al., 2015; Brugnolo et al., 2019) and has been recommended for differential diagnosis of AD especially in clinically ambiguous or early cases by the Delphi consensus of the European Association of Nuclear Medicine (EANM) and the European Academy of Neurology (EAN; Nobili et al., 2018).

To support the clinical diagnosis, a biomarker-based diagnostic scheme has been proposed for AD including categorization for amyloid, tau, and existing neuronal degeneration (ATN scheme; Jack and Holtzman, 2013; Jack et al., 2016, 2018). Neuronal degeneration in AD can be assessed by cranial magnetic resonance imaging (MRI) or FDG-PET (Jack et al., 2016), often presenting with asymmetric patterns. Hippocampal atrophy has been predominantly found in the left hemisphere in subjects with mild cognitive impairment (MCI) and AD (Shi et al., 2009) and asymmetry of the hippocampus, amygdala, caudate and cortex was predictive of disease progression from MCI to AD (Wachinger et al., 2016) and associated with AD-related single nucleotide polymorphisms (Wachinger et al., 2018). Asymmetric neuronal degeneration is correlated with asymmetries of amyloid burden, both concordant with lateralized cognitive symptoms (Frings et al., 2015). However, only a few studies are dealing with the clinical impact of asymmetric neuronal degeneration patterns.

The “Consortium to Establish a Registry for Alzheimer’s disease” (CERAD) test battery is a widespread and well recognized clinical neuropsychological testing method for determining AD (Welsh et al., 1994). Total CERAD scores, published by Chandler et al. (2005) also allow judging the severity of AD, which is an advantage over screening methods like Mini-Mental-State-Examination (MMSE; Chandler et al., 2005; Ehrensperger et al., 2010; Wolfsgruber et al., 2014). We previously found that FDG-PET abnormalities in AD-typical brain regions and CERAD total scores are well associated with clinical AD (Beyer et al., 2019). Earlier results indicated that individual scores of the CERAD battery provide a good representation of the left-hemispheric dysfunction in AD patients but impairment of the right hemisphere appears to be only poorly represented (Teipel et al., 2006).

This study aimed to evaluate the impact of lateralized neuronal degeneration on the detection of AD by detailed clinical testing with the integration of the timeline of clinical progression. Furthermore, we compared associations between clinical evaluation and lateralized neuronal degeneration between FDG-PET and hippocampal atrophy to structural MRI. Finally, we investigated if specific subtests of the CERAD battery show associations with lateralized neuronal degeneration.

## Materials and Methods

### Study Design Patient Enrolment

The study included 146 patients with a clinical diagnosis of AD. All suspected AD cases were confirmed in clinical follow-up (23.7 ± 13.8 months) and a subset of *n* = 49 received repeated neuropsychological testing including the CERAD test battery. The subjects were recruited and scanned in a clinical setting at the University of Munich (Department of Nuclear Medicine) between 2010 and 2016. Patients had been referred by the Departments of Neurology, Psychiatry, and Institute for Stroke and Dementia Research. The majority of patients were recruited *via* specialized outpatient clinics. Data analysis was approved by the local ethics committee (19-004). All subjects underwent clinical dementia workup, including detailed cognitive testing and FDG-PET. Data on handedness was available in 104/146 patients.

### Clinical Assessment and Cognitive Testing

Neurological examination and neuropsychological testing were performed including the CERAD battery plus Trail-Making Test A and B and verbal fluency tests (CERAD+; Morris et al., 1989), resulting in raw values and *Z*-scores for all subtests. We created a total CERAD score by summing up the raw values from the individual CERAD subtests by inclusion of subtests as described earlier (Chandler et al., 2005; Beyer et al., 2019): verbal fluency (maximum score = 24), modified BNT (maximum score = 15), word list learning (maximum score = 30), constructional praxis (maximum score = 11), word list recall (maximum score = 10), word list recognition discriminability (maximum score = 10). Thus, the maximum achievable score was 100 (Chandler et al., 2005). For patients with clinical follow-up examination, the annual change of total CERAD was calculated. Age, gender, and years of education (YoE) were obtained as covariates.

### MRI

MRI (1.5/3.0 Tesla magnets) using a T1w sequence for hippocampal atrophy evaluation was available from 96/146 included patients. The Scheltens scale, a score for medial temporal lobe atrophy which ranges from 0 to 4, was rated visually by an expert in Radiology (Minoshima et al., 1995). A summed score was built for both hippocampi and asymmetry was quantified as the difference between left and right scoring.

### FDG-PET

#### FDG-PET Acquisition

FDG was purchased commercially. All FDG-PET images were created using either a 3-dimensional GE Discovery 690 PET/CT scanner or a Siemens ECAT EXACT HR+ PET scanner. Each subject fasted for at least 6 h, resulting in a plasma glucose level less than 120 mg/dl (6.7 mM) at the time of the tracer administration. All patients were injected i.v. with a dose of 142 ± 8 MBq FDG as a slow bolus while sitting quietly in a room with a low noise level and dimmed light. A static emission frame was acquired from 30 to 60 min p.i. for the Siemens ECAT EXACT HR+ PET scanner, respectively from 30 to 45 min p.i. for the Discovery 690 PET/DT. For attenuation correction, a transmission scan with external 68Ge-sources (Siemens) or a low-dose CT was performed before the static acquisition. PET data were reconstructed iteratively (GE) or with filtered back-projection (Siemens).

#### Visual Analysis of FDG-PET

Three-dimensional stereotactic surface projections (3D-SSP; Minoshima et al., 1995) were created using the software Neurostat (Department of Radiology, University of Washington, Seattle, WA, USA) for visual image interpretation. Visual assessment of the 3D-SSP images was carried out by an expert in Nuclear Medicine using tracer uptake and *Z*-score maps (global mean scaling). Voxel-wise *Z-scores* were calculated in Neurostat by comparing the individual tracer uptake to historical FDG-PET images from a healthy age-matched cohort (*n* = 18). The reader had access to clinical information and structural imaging. A simplified approach of the *t-sum* method published by Herholz et al. (2002) was performed for visual quantification as previously published (Beyer et al., 2019). In brief, the rating was grouped into four grades of neuronal degeneration ranging from 0 to 3, where 0 is no neuronal degeneration, 1 is mild neuronal degeneration, 2 is moderate neuronal degeneration, and 3 is severe neuronal degeneration. Preselected AD-typical regions in FDG-PET were graded: the bilateral parietal lobe, bilateral temporal lobe, and bilateral posterior cingulate cortex. Summed scores were calculated for the whole brain and each hemisphere (scores 0 to 9). Asymmetry was calculated as the difference between the left and right hemisphere or subregion (left-right). A subset of half of the patients (*n* = 73) was rated twice by the first reader and by one additional expert in Nuclear Medicine to test for intra-/inter-rater reliability.

#### Semiquantitative Analysis of FDG-PET

Also, semiquantitative analysis of FDG-PET was conducted. The images have been anonymized before analysis. PMOD software (version 3.5, PMOD Technologies Limited, Zürich, Switzerland) was used for coregistration of all individual FDG-PET image volumes to an in-house FDG-PET template within the MNI space (Daerr et al., 2017). In analogy to the visual analysis, we measured the mean activity within bilateral parietal, bilateral temporal, and bilateral posterior cingulate cortex volumes of interest (VOIs) of the Hammers atlas (Hammers et al., 2003) and scaled the measured regional activities by a cerebellum reference region to generate standardized uptake value ratios (SUVr). Asymmetry was calculated by the Asymmetry-Index [AI = (left − right)/(left + right)*100] for hemispheres and subregions.

### Calculations and Statistical Analysis

Mean visual ratings, semiquantitative results, and mean asymmetries were calculated for each hemisphere and subregions (parietal, temporal, and posterior cingulate cortex). Intra-/inter-rater agreement were assessed by intra-/inter-class correlation coefficients. Visual ratings and semiquantitative results of both hemispheres and separately for the left/right hemisphere were correlated using Pearson’s correlation coefficient. FDG-PET results were compared between sides using a Wilcoxon test for visual and two-sided *t*-test for semiquantitative analysis. Mean asymmetry was compared between the whole hemisphere and subregions by a one-way ANOVA. Asymmetry indices of all cortical regions were correlated with each other and the total asymmetry index using a Pearson’s correlation coefficient.

Cognitive performance was correlated with the visual rating using Spearman–Rho and with the semiquantitative analysis using Pearson’s correlation coefficient. Multiple regression analyses were conducted with the cognition (expressed with CERAD) as the dependent and (i) asymmetry as the independent variable or (ii) Scheltens-Scale as the independent variable, both with age, gender, years of education, and the total hypometabolism in FDG-PET (sum score of both hemispheres) as covariates for both visual and semiquantitative analyses.

For separate analysis of both right- and left-asymmetric hypometabolism, patients with (nearly) unilateral neuronal degeneration by FDG-PET (≥2.0 vs. <2.0 for the contralateral side in the visual rating) were selected, and both groups were separately correlated with the CERAD total score using Spearman–Rho (visual) or Pearson’s correlation coefficient (semiquantitative).

Based on the change of the total CERAD score per year and patient and the CERAD difference between correlation lines of right- and left-lateralized cases for the full range of observed neuronal degeneration, we calculated an average detection gap between the left and right hemisphere for both visual and semiquantitative FDG-PET results as following: the mean CERAD was calculated for visual/semiquantitative FDG-PET results separately for left- und right-asymmetric cases using the mean visual/semiquantitative FDG-PET results with the formula of all four correlations, respectively. The mean CERAD difference between left- and right-asymmetric cases was then divided by the mean annual decrease in CERAD of all available follow-up cases and compared against each other.

Additional regression analyses were performed using the left-/right-hemispheric hypometabolism as the dependent and all CERAD subscores separately as the independent variables with age, gender, and education as covariates controlled for multiple testing with Bonferroni correction.

All statistical analyses were performed using SPSS (version 25.0, IBM, Armonk, New York, NY, USA) and a significance level of *p* < 0.05 was applied in all analyses.

## Results

### Demographics and Asymmetry in FDG Uptake

The study population consisted of 146 subjects (57.5% female) with a clinical diagnosis of AD (follow-up 23.7 ± 13.8 months) and available FDG-PET data. Of all patients, six patients were diagnosed with atypical AD, 133 patients with typical AD (*n* = 41 early-onset AD, *n* = 92 late-onset AD), and seven patients were not further specified. 90/104 patients (86.5%) were right-handed, 6/104 (5.8%) were left-handed and 8/104 (7.7%) claimed to be ambidextrous. For details of the study population, see Table 1.

**Table 1:** Demographic and clinical data of the study population (*n* = 146).

Gender (♂/♀)	♂62/♀84
Age (y ±SD)	70.5 ± 8.0
Education (y ±SD)	12.4 ± 3.2
Handedness (*n* = 104)	90 right, 16 left, 8 ambidextrous
Total CERAD score (±SD)	54.3 ± 13.3
CERAD subscores (±SD)	
Verbal fluency (animals)	12.8 ± 5.4
Modified BNT	12.3 ± 2.6
MMSE (±SD)	22.7 ± 4.1
Word list learning total	11.3 ± 4.6
Word list learning 1	2.5 ± 1.5
Word list learning 2	3.8 ± 1.7
Word list learning 3	4.8 ± 1.7
Word list recall	2.1 ± 1.9
Word list intrusion	1.7 ± 2.3
Word list savings	42.1 ± 33.1
Word list recognition-discriminability (%)	82.5 ± 13.5
Constructional praxis	9.1 ± 2.3
Constructional recall	3.3 ± 3.0
Savings figures	30.0 ± 28.0
CERAD+ scores (±SD)	
Verbal fluency (S-words)	9.2 ± 5.0
TMT-A (s)	85.9 ± 40.6
TMT-B (s)	185.6 ± 85.3
TMT A/B (s)	2.9 ± 1.1
Clinical follow-up (±SD)	23.7 ± 13.8
Total CERAD score follow up (±SD, *n* = 49)	51.5 ± 13.7

*SD, standard deviation; MMSE, mini-mental-state-examination; CERAD, Consortium to Establish a Registry for Alzheimer’s disease; BNT, Boston Naming Test; TMT, Trail Making Test; y, years; m, months; s, seconds*.

Asymmetries in FDG uptake were assessed by visual and semiquantitative measures (see Table 2). Intra- and inter-rater agreement was excellent (intra-rater: κ = 0.92 *p* < 0.001, inter-rater: κ = 0.96 *p* < 0.001). Visual ratings and semiquantitative results significantly correlated for both hemispheres (*R* = 0.583, *p* < 0.001) and separately for the left (*R* = 0.700, *p* < 0.001) and right hemisphere (*R* = 0.707, *p* < 0.001). Visual analysis showed a more pronounced hypometabolism pattern of neuronal degeneration in the left hemisphere (*p* < 0.001). Asymmetry was pronounced in the posterior cingulate cortex (*p* < 0.001), followed by the parietal lobe (*p* = 0.033) and less prominent in the temporal lobe (*p* = 0.059). The semiquantitative analysis also indicated significant differences between left and right for the posterior cingulate cortex (*p* < 0.001) and the parietal cortex (*p* < 0.001) but not for the full hemisphere (*p* = 0.122) or the temporal cortex (*p* = 0.907). ANOVA confirmed these results, revealing a significant difference between asymmetry magnitude in subregions and full hemispheres for semiquantitative assessment (*F*_(146,3)_ = 6.964; *p* < 0.001). *Post hoc* testing indicated stronger asymmetry in the posterior cingulate cortex when compared to the lateral parietal lobe (*p* = 0.040), the temporal lobe (*p* < 0.001) and entire hemispheres (*p* = 0.003). Asymmetry indices of all cortical subregions significantly correlated with each other and the total asymmetry index (visual: all *R* > 0.584, all *p* < 0.01; semiquantitative: all *R* > 0.573, all *p* < 0.01).

**Table 2:** Visual and semiquantitative FDG-PET results and asymmetries.

Region		Visual FDG-PET (3DSSP-rating)	Visual asymmetries (left-right)	Semiquantitative FDG-PET (SUVr)	Semiquantitative asymmetries (AI)
Hemispheres (±SD)	Right	−3.1 ± 2.3	−0.7 ± 2.5	0.85 ± 0.07	−1.04 ± 3.63
	Left	−3.8 ± 2.2		0.83 ± 0.07	
Parietal cortex (±SD)	Right	−1.2 ± 1.0	−0.2 ± 1.0	0.87 ± 0.08	−1.33 ± 3.52
	Left	−1.4 ± 1.0		0.85 ± 0.08	
Temporal cortex (±SD)	Right	−0.7 ± 0.9	−0.2 ± 1.1	0.81 ± 0.07	−0.69 ± 4.14
	Left	−1.0 ± 0.9		0.82 ± 0.07	
Posterior cingulate cortex (±SD)	Right	−1.2 ± 0.9	−0.4 ± 0.7	0.89 ± 0.09	−2.44 ± 2.29
	Left	−1.6 ± 0.8		0.85 ± 0.08	

*SD, standard deviation; FDG, F-18-fluorodesoxyglucose; PET, positron-emission-tomography; 3DSSP, three-dimensional stereotactic surface projections; SUVr, standardized uptake value ratios; AI, asymmetry index*.

### Asymmetry in FDG-PET as an Independent Predictor of Cognitive Impairment

**Figure 1:**
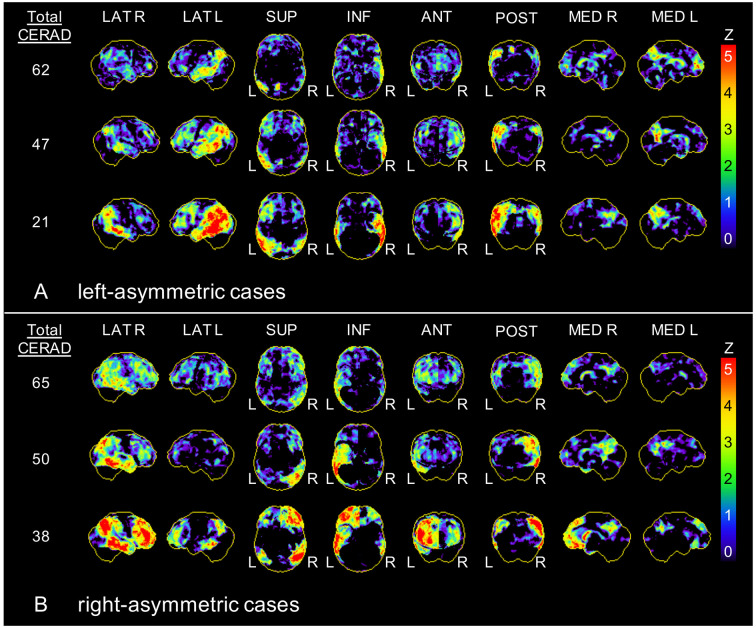
Exemplary asymmetric hypometabolism patterns. Three-dimensional stereotactic surface projections (*Z*-score maps) of patients with **(A)** left-asymmetric and **(B)** right-asymmetric hypometabolism in F-18-fluorodeoxyglucose positron emission tomography (FDG-PET). CERAD, Consortium to establish a registry for Alzheimer’s disease; R, right; L, left; LAT, lateral; SUP, superior; INF, inferior; ANT, anterior; POST, posterior; MED, medial.

Our recent findings of a significant correlation between neuronal degeneration and cognitive impairment (Beyer et al., 2019) were confirmed in the current subset and showed significant associations between deteriorated FDG-PET in bihemispheric AD target regions and the CERAD total score (visual: ρ = 0.435; *p* < 0.001; semiquantitative: *R* = 0.442, *p* < 0.001). Using a multiple regression analysis, asymmetry in FDG-PET was a significant predictor of current cognitive impairment (visual: *β* = −0.288, *p* < 0.001; semiquantitative: *β* = −0.451, *p* < 0.001) and explained 35.4% (visual)/32.0% (semiquantitative) of the variance in CERAD, independent from age, gender, education and total burden of neuronal degeneration to FDG-PET (see Table 3). Left-lateralized neuronal degeneration was associated with stronger presence of cognitive impairment. Exemplary patients with left- and right-asymmetric neuronal degeneration in FDG-PET 3D-SSP are visualized in Figure 1.

**Table 3:** Multiple regression analysis for asymmetry in the visual and semiquantitative analysis based on total CERAD score.

Region	Independent variable Asymmetry β (*p*)	Covariates Age β (*p*)	Gender β (*p*)	YoE β (*p*)	Total burden FDG-PET β (*p*)
Visual
Hemispheres	−0.288 (<0.001)	−0.168 (0.020)	0.177 (0.017)	0.220 (0.002)	0.435 (<0.001)
Parietal lobe	−0.276 (<0.001)	−0.163 (0.024)	0.168 (0.023)	0.211 (0.004)	0.440 (<0.001)
Temporal lobe	−0.262 (<0.001)	−0.181 (0.013)	0.161 (0.029)	0.231 (0.002)	0.429 (<0.001)
Posterior cingulate cortex	−0.219 (0.003)	−0.149 (0.045)	0.158 (0.037)	0.238 (0.001)	0.414 (<0.001)
Semiquantitative					
Hemispheres	−0.451 (<0.001)	−0.140 (0.049)	0.131 (0.071)	0.185 (0.012)	0.442 (<0.001)
Parietal lobe	−0.419 (<0.001)	−0.125 (0.081)	0.110 (0.129)	0.191 (0.010)	0.414 (<0.001)
Temporal lobe	−0.445 (<0.001)	−0.143 (0.046)	0.139 (0.057)	0.191 (0.010)	0.444 (<0.001)
Posterior cingulate cortex	−0.168 (0.035)	−0.089 (0.251)	0.069 (0.376)	0.269 (0.001)	0.303 (<0.001)

*CERAD, Consortium to Establish a Registry for Alzheimer’s disease. FDG-PET, F-18-fluorodeoxyglucose positron emission tomography*.

In contrast to FDG-PET, asymmetry of neuronal degeneration in the hippocampus to MRI as rated by Scheltens Scale (asymmetry: left-right) did not indicate an independent prediction of the current cognition when controlled for age, gender, education, and total semiquantitative burden of neuronal degeneration to FDG-PET (*β* = −0.034; *p* = 0.731).

### Delayed CERAD Detection of Right-Hemispheric Neuronal Degeneration

Next, we aimed to investigate associations of neuronal degeneration and cognitive decline independently for FDG-PET results of each hemisphere. Considering all 146 cases, we observed a stronger correlation for neuronal degeneration in the left hemisphere with the total CERAD score (visual: ρ = −0.479, *p* < 0.001, semiquantitative: *R* = 0.497, *p* < 0.001) when compared to neuronal degeneration in the right hemisphere (visual: ρ = −0.205, *p* = 0.013; semiquantitative: *R* = 0.282, *p* = 0.001).

**Figure 2:**
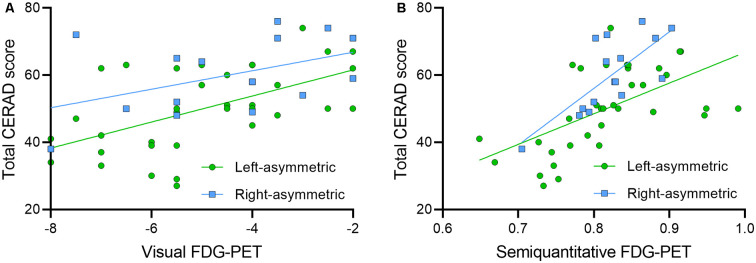
Comparison of cases with dominant right- and left-lateralization. Correlation plots show the functions of **(A)** visual FDG-PET and **(B)** semiquantitative FDG-PET with total CERAD score for left- (*n* = 35, green dots) and right- (*n* = 16, blue squares) asymmetric cases.

To increase specificity for the side of affection, we defined asymmetric cases with (nearly) unilateral neuronal degeneration by FDG-PET (≥2.0 vs. <2.0 for the contralateral side in the visual rating) and were able to compare 35 cases predominantly affected on the left hemisphere and 16 cases predominantly affected on the right hemisphere. Both left-hemispheric (visual: ρ = 0.571, *p* < 0.001; semiquantitative: *R* = 0.575, *p* < 0.001) and right-hemispheric (visual: ρ = 0.463, *p* = 0.071; semiquantitative: *R* = 0.740, *p* = 0.001) neuronal degeneration was associated significantly with the total CERAD score. However, the function of total CERAD score and right-lateralized neuronal degeneration ranged at a higher level when compared to the function of CERAD and left-lateralized neuronal degeneration (see Figure 2).

The averaged CERAD score as a function of FDG-PET of left dominant cases ranged 9.9 CERAD score units below the average of right dominant cases. Considering longitudinal CERAD data of 49 subjects of this cohort, the total CERAD score deteriorated by −3.6 ± 7.4 points per year. This hypothetical model results in an average detection gap of 2.7 years for CERAD assessment in right-lateralized cases when compared to left-lateralized cases at an equal neuronal degeneration level.

### Associations of Right-Hemispheric Neuronal Degeneration With CERAD Subscores

Finally, we tested if CERAD and CERAD+ subscores may detect right-lateralized neuronal degeneration better when compared to the total CERAD score. Right-hemispheric neuronal degeneration in visual and semiquantitative analysis was significantly associated only with MMSE (semiquantitative: *β* = 0.429, *p* < 0.001) and constructional praxis (semiquantitative: *β* = 0.292, *p* = 0.008), whereas left-hemispheric hypometabolism significantly correlated with 8/18 subscores. For details of all correlations with the visual rating and semiquantitative results see Table 4.

**Table 4:** Correlation of CERAD subscores with hypometabolism in FDG-PET.

Subscore	Visual		Semiquantitative	
	Left *β* (*p*)	Right *β* (*p*)	Left *β* (*p*)	Right *β* (*p*)
Verbal fluency (animals)	0.466 (<0.001)	0.142 (n.s.)	0.389 (<0.001)	0.177 (n.s.)
Modified BNT	0.262 (0.018)	0.037 (n.s.)	0.349 (<0.001)	0.194 (n.s.)
MMSE	0.520 (<0.001)	0.297 (0.018)	0.542 (<0.001)	0.429 (<0.001)
Word list learning total	0.460 (<0.001)	0.159 (n.s.)	0.446 (<0.001)	0.240 (n.s.)
Word list learning 1	0.393 (<0.001)	0.121 (n.s.)	0.437 (<0.001)	0.273 (n.s.)
Word list learning 2	0.398 (<0.001)	0.204 (n.s.)	0.339 (0.004)	0.219 (n.s.)
Word list learning 3	0.307 (0.018)	0.274 (n.s.)	0.257 (n.s.)	0.247 (n.s.)
Word list recall	0.248 (n.s.)	0.208 (n.s.)	0.130 (n.s.)	0.093 (n.s.)
Word list intrusion	0.012 (n.s.)	0.098 (n.s.)	0.031 (n.s.)	0.053 (n.s.)
Word list savings	-0.010 (n.s.)	0.185 (n.s.)	-0.103 (n.s.)	0.043 (n.s.)
Word list recognition—discriminability	0.094 (n.s.)	0.125 (n.s.)	0.088 (n.s.)	0.115 (n.s.)
Constructional praxis	0.202 (n.s.)	0.180 (n.s.)	0.259 (0.018)	0.292 (0.008)
Constructional recall	0.209 (n.s.)	0.183 (n.s.)	0.229 (n.s.)	0.217 (n.s.)
Savings figures	0.163 (n.s.)	0.165 (n.s.)	0.158 (n.s.)	0.167 (n.s.)
Verbal fluency (S-words)	0.287 (0.008)	0.004 (n.s.)	0.310 (0.003)	0.089 (n.s.)
TMT A	0.232 (n.s.)	0.162 (n.s.)	0.252 (n.s.)	0.232 (n.s.)
TMT B	0.331 (n.s.)	0.210 (n.s.)	0.152 (n.s.)	0.100 (n.s.)
TMT A/B	0.185 (n.s.)	-0.046 (n.s.)	0.011 (n.s.)	-0.088 (n.s.)

*CERAD, Consortium to Establish a Registry for Alzheimer’s disease; FDG, F-18-fluorodeoxyglucose; PET, positron-emission-tomography; BNT, Boston Naming Test; MMSE, mini-mental state examination; TMT, Trail Making Test; n.s., non-significant*.

## Discussion

In the present study, we investigated the relationship between asymmetric neuronal degeneration patterns measured by FDG-PET and cognition expressed by the CERAD test battery in clinical AD patients. We demonstrate that asymmetries in FDG-PET constitute an independent predictor of current cognitive impairment. Predominant left-hemispheric neuronal degeneration led to more severe cognitive impairment as assessed by the CERAD battery when compared to right-hemispheric neuronal degeneration. In contrast to the left hemisphere, right-hemispheric cognitive abilities are poorly represented in CERAD subscores, with only two subscores showing a significant association with right-hemispheric neuronal degeneration.

### Associations of Asymmetric Neuronal Degeneration and Cognitive Function

Asymmetries of brain function are well known and the reasons for asymmetric neuronal degeneration expressed by gray matter asymmetries in aging and neurodegenerative diseases are being discussed extensively (Minkova et al., 2017). Previous smaller scaled studies showed a predominance of left hemisphere metabolic dysfunction in MCI and AD (Loewenstein et al., 1989; Murayama et al., 2016). In line with this, we found a left dominant neuronal degeneration pattern in our AD cohort when compared to the right hemisphere and also separately in distinct brain regions known to be affected in AD (parietal, temporal, and posterior cingulate cortex). Asymmetry indices of all regions significantly correlated with each other, indicating that left-/ right-asymmetric asymmetry is concordant between regions on a single-patient level.

Asymmetry in FDG-PET proved to be an independent predictor of cognitive function, indicating that left-hemispheric hypometabolism is associated with a stronger presence of cognitive impairment in clinical testing. It has already been shown in patients with MCI and AD that the left-dominant group in FDG-PET had lower scores in verbal memory and a greater tendency to be diagnosed with AD rather than MCI (Murayama et al., 2016). Thus, the consistent findings in the current cohort established the basis to study the impact of asymmetry on FDG-PET evaluation in a clinical setting.

Asymmetric hippocampal atrophy in MRI rated by the Scheltens Scale did not indicate an independent prediction of cognition. We focused on a visual approach for a rating of hippocampal atrophy to generate results that can be transferred into a clinical routine setting. However, quantitative approaches with segmentation and measurement methods analyzing hippocampal subfields (Giuliano et al., 2017) and/or cortical regions could probably improve the informative value of asymmetry in MRI.

### Detection Gap Between Left- and Right-Asymmetric Cases

In further disease progression, cognitive deterioration correlates with neurodegeneration biomarkers (Beyer et al., 2019) with greater cognitive impairment along the continuum from normal cognitive status to MCI and AD dementia. Asymmetries of neuronal degeneration patterns and their influence on cognition have not been considered in the diagnostic workup so far (Jack et al., 2010, 2013). It has been hypothesized that early stages of AD may be characterized by a lateralized pattern with left-hemispheric hypometabolism and decreasing differences with further disease progression (Weise et al., 2018). Also, patients with left-hemispheric dominance of neuronal degeneration are more likely to be diagnosed with MCI (Cherbuin et al., 2010), which might lead to an assignment bias in our cohort.

The reason for different neuronal degeneration patterns has not been clarified yet, but we demonstrate that left-asymmetric cases might be clinically detectable earlier than right-asymmetric cases. We found a detection gap of >2.5 years of a potential later-diagnosis of right-asymmetric patients compared to left-asymmetric patients when using the CERAD test battery. Clinical symptoms deriving from left-hemispheric neuronal degeneration seem to be stronger represented by the CERAD battery. Consequently, left- vs. right-asymmetric neuronal degeneration patterns in FDG-PET need to be weighted differently because right-asymmetric neuronal degeneration might be insufficiently represented in clinical testing.

### Representation of Asymmetric Neuronal Degeneration by CERAD Subscores

Several studies have shown that different deteriorated brain subdomains are linked to either left- or right-hemispheric neuronal degeneration. Verbal and nonverbal impairments (Zahn et al., 2004), severe dysfunction in verbal memory, general memory, and delay recall (Murayama et al., 2016), language and visuospatial deficits (Haxby et al., 1985) were associated with left-hemispheric neuronal degeneration. Right-hemispheric neuronal degeneration was associated with constructional praxis recall (Han et al., 2015) and drawing test performance (Förster et al., 2010) and, together with left-hemispheric alterations, with constructional praxis (Han et al., 2015).

For the clinical representation of the existing neuronal degeneration, the CERAD battery has been proven to adequately represent left-hemispheric dysfunction in AD, but with difficulties displaying right-hemispheric dysfunction (Welsh et al., 1994; Teipel et al., 2006). This matches our finding that despite the higher correlation of the CERAD total score with left-hemispheric hypometabolism compared to right-hemispheric hypometabolism, more CERAD subscores significantly correlated with left-, and only two correlated with right-hemispheric hypometabolism. Only the MMSE and constructional praxis subtests were found to be significantly associated with right-hemispheric neuronal degeneration. Therefore, subjects with clinical apraxia and even mild to moderate cognitive impairment (as assessed by MMSE) should be considered for FDG-PET to search for right-hemispheric neuronal degeneration.

Some studies found symmetric associations with CERAD scores (Schönknecht et al., 2011) and clusters with the involvement of both hemispheres (Staffaroni et al., 2017). Many cognitive abilities are known to derive from both left- and right-hemispheric brain areas and disruptions in brain activities in some regions of the brain consequently lead to worsening of other connected functions as well. In line with this, the constructional praxis subtest was significantly associated with the right-, but also with left-hemispheric neuronal degeneration. Also, the MMSE as a global measurement of cognitive function is significantly associated with both hemispheres. Whereas more specific subtests are available for left-hemispheric abilities, the clinical representation of the right-hemispheric neuronal degeneration is limited to these two subtests.

Additional tests included in the CERAD+ test battery (e.g., TMT, verbal fluency with S-words) as well do not sufficiently represent right-hemispheric brain functions as the represented abilities also involve both hemispheres. Furthermore, it has been pointed out that some subscores like BNT, word list recognition, or constructional praxis and recall tests should be considered less strongly for early detection of dementia because of strong ceiling effects (Luck et al., 2018).

Therefore, the early and accurate clinical assessment of right-asymmetric cases remains challenging, and additional neuropsychological testing (especially with behavioral measures) should be considered especially in cases with cognitive impairments of right-hemispheric brain functions as they might be poorly represented by CERAD. Furthermore, imaging with FDG-PET can also be considered to support clinical diagnosis.

### Limitations

Among the limitations of our study, we note that correlations with neuropsychological testing are limited to CERAD test battery results. Other neuropsychological tests and behavioral measures are not included in this study and analysis of additional parameters would be of interest. While *p*-tau in the cerebrospinal fluid was available and pathologic in all of our cases, we did not have a comprehensive evaluation of Aβ in all cases. Therefore, the ATN scheme (Jack et al., 2016) could not be fulfilled with the proposed biomarkers. However, the diagnoses of AD were confirmed by long-term clinical follow-up, and only subjects with a confident clinical diagnosis of AD were included in the analysis. We focused on covariates recommended to support the diagnosis of AD, but we could not cover the full range of environmental factors, comorbidities, and apolipoprotein E status which otherwise could have influenced our results. A major strength of the study lies in the clinical setting to guarantee transferability into routine clinical scenarios.

A small subset of patients in this sample was classified as atypical AD. Asymmetry analysis in this subsample would be of great interest but was not possible due to the limited amount of cases. Data on handedness was only available in a subset of patients (*n* = 104) and the sample size of left-handed subjects was too small for separate analysis. Therefore, the potential effect of the difference between the dominant and minor hemisphere cannot be further investigated and might have influenced the results.

## Conclusion

Asymmetry in FDG-PET predicts cognitive performance, but only left-hemispheric neuronal degeneration is well represented by the clinical used CERAD battery. Therefore, clinical diagnosis in patients with right-hemispheric neuronal degeneration might be delayed, calling for extended neuropsychological testing and additional diagnostic procedures in clinically unclear cases to capture cognitive impairments related to right-hemispheric neuronal degeneration.

## Data Availability Statement

The raw data supporting the conclusions of this article will be made available by the authors, upon reasonable request.

## Ethics Statement

The studies involving human participants were reviewed and approved by LMU Munich ethics commitee (19-004). Written informed consent for this retrospective analysis was not required in accordance with the national legislation and the institutional requirements.

## Author Contributions

AK: acquisition of data, analysis, interpretation of data, document writing, and editing. JS, MS, AS, NA, PB, and AR: acquisition of data, interpretation of molecular imaging data, and revising of the manuscript. SS, CPr, EW, SL, CC, DJ, OP, CPa, RP, AD, KB, JL, and AZ: acquisition of clinical patient data, drafting and revising of the manuscript. PK and MU: acquisition of radiological data, drafting and revising of the manuscript. MB: interpretation of molecular imaging data, conception, intellectual input, drafting and revising of the manuscript. LB: conception and design, acquisition of data, document editing, final manuscript approval for submission and publication. All authors contributed to the article and approved the submitted version.

## Conflict of Interest

RP is on the advisory board for Biogen, has consulted for Eli Lilly, is a grant recipient from Janssen Pharmaceutica and Boehringer Ingelheim, and has received speaker honoraria from Janssen-Cilag, Pfizer, and Biogen. PB declares a research collaboration with GE. AR received speaker honoraria from Piramal Imaging and GE Healthcare. MB received speaker honoraria from GE healthcare and LMI and is an advisor of LMI. The remaining authors declare that the research was conducted in the absence of any commercial or financial relationships that could be construed as a potential conflict of interest.

## References

[B1] Beyer L. Schnabel J. Kazmierczak P. Ewers M. Schönecker S. Prix C.. (2019). Neuronal injury biomarkers for assessment of the individual cognitive reserve in clinically suspected Alzheimer’s disease. Neuroimage Clin. 24:101949. 10.1016/j.nicl.2019.101949 31398553 PMC6699250

[B2] Brugnolo A. De Carli F. Pagani M. Morbelli S. Jonsson C. Chincarini A.. (2019). Head-to-head comparison among semi-quantification tools of brain FDG-PET to aid the diagnosis of prodromal Alzheimer’s disease. J. Alzheimers Dis. 68, 383–394. 10.3233/JAD-181022 30776000

[B3] Chandler M. J. Lacritz L. H. Hynan L. S. Barnard H. D. Allen G. Deschner M.. (2005). A total score for the CERAD neuropsychological battery. Neurology 65, 102–106. 10.1212/01.wnl.0000167607.63000.38 16009893

[B4] Cherbuin N. Réglade-Meslin C. Kumar R. Sachdev P. Anstey K. J. (2010). Mild cognitive disorders are associated with different patterns of brain asymmetry than normal aging: the PATH through life study. Front. Psychiatry 1:11. 10.3389/fpsyt.2010.00011 21423423 PMC3059654

[B5] Colucci L. Bosco M. Fasanaro A. M. Gaeta G. L. Ricci G. Amenta F. (2014). Alzheimer’s disease costs: what we know and what we should take into account. J. Alzheimers Dis. 42, 1311–1324. 10.3233/JAD-131556 25024334

[B6] Daerr S. Brendel M. Zach C. Mille E. Schilling D. Zacherl M. J.. (2017). Evaluation of early-phase [^18^F]-florbetaben PET acquisition in clinical routine cases. Neuroimage Clin. 14, 77–86. 10.1016/j.nicl.2016.10.005 28138429 PMC5257027

[B7] De Deyn P. P. Goeman J. Vervaet A. Dourcy-Belle-Rose B. Van Dam D. Geerts E. (2011). Prevalence and incidence of dementia among 75-80-year-old community-dwelling elderly in different districts of Antwerp, Belgium: The Antwerp Cognition (ANCOG) Study. Clin. Neurol. Neurosurg. 113, 736–745. 10.1016/j.clineuro.2011.07.030 21862210

[B8] Ehrensperger M. M. Berres M. Taylor K. I. Monsch A. U. (2010). Early detection of Alzheimer’s disease with a total score of the German CERAD. J. Int. Neuropsychol. Soc. 16, 910–920. 10.1017/S1355617710000822 20682088

[B9] Förster S. Teipel S. Zach C. Rominger A. Cumming P. Fougere C. L.. (2010). FDG-PET mapping the brain substrates of visuo-constructive processing in Alzheimer’s disease. J. Psychiatr. Res. 44, 462–469. 10.1016/j.jpsychires.2009.09.012 19875130

[B10] Frings L. Hellwig S. Spehl T. S. Bormann T. Buchert R. Vach W.. (2015). Asymmetries of amyloid-β burden and neuronal dysfunction are positively correlated in Alzheimer’s disease. Brain 138, 3089–3099. 10.1093/brain/awv229 26280595

[B11] Giuliano A. Donatelli G. Cosottini M. Tosetti M. Retico A. Fantacci M. E. (2017). Hippocampal subfields at ultra high field MRI: an overview of segmentation and measurement methods. Hippocampus 27, 481–494. 10.1002/hipo.22717 28188659 PMC5573987

[B12] Hammers A. Allom R. Koepp M. J. Free S. L. Myers R. Lemieux L.. (2003). Three-dimensional maximum probability atlas of the human brain, with particular reference to the temporal lobe. Hum. Brain Mapp. 19, 224–247. 10.1002/hbm.10123 12874777 PMC6871794

[B13] Han J. Y. Byun M. S. Seo E. H. Yi D. Choe Y. M. Sohn B. K.. (2015). Functional neural correlates of figure copy and recall task performances in cognitively impaired individuals: an ^18^F-FDG-PET study. NeuroReport 26, 1077–1082. 10.1097/WNR.0000000000000476 26509549

[B14] Haxby J. V. Duara R. Grady C. L. Cutler N. R. Rapoport S. I. (1985). Relations between neuropsychological and cerebral metabolic asymmetries in early Alzheimer’s disease. J. Cereb. Blood Flow Metab. 5, 193–200. 10.1038/jcbfm.1985.25 3988821

[B15] Herholz K. Salmon E. Perani D. Baron J. C. Holthoff V. Frölich L.. (2002). Discrimination between Alzheimer dementia and controls by automated analysis of multicenter FDG-PET. NeuroImage 17, 302–316. 10.1006/nimg.2002.1208 12482085

[B17] Jack C. R. Jr. Bennett D. A. Blennow K. Carrillo M. C. Dunn B. Haeberlein S. B.. (2018). NIA-AA research framework: toward a biological definition of Alzheimer’s disease. Alzheimers Dement. 14, 535–562. 10.1016/j.jalz.2018.02.018 29653606 PMC5958625

[B18] Jack C. R. Jr. Bennett D. A. Blennow K. Carrillo M. C. Feldman H. H. Frisoni G. B.. (2016). A/T/N: an unbiased descriptive classification scheme for Alzheimer disease biomarkers. Neurology 87, 539–547. 10.1212/WNL.0000000000002923 27371494 PMC4970664

[B16] Jack C. R. Jr. Holtzman D. M. (2013). Biomarker modeling of Alzheimer’s disease. Neuron 80, 1347–1358. 10.1016/j.neuron.2013.12.003 24360540 PMC3928967

[B19] Jack C. R. Jr. Knopman D. S. Jagust W. J. Petersen R. C. Weiner M. W. Aisen P. S.. (2013). Tracking pathophysiological processes in Alzheimer’s disease: an updated hypothetical model of dynamic biomarkers. Lancet Neurol. 12, 207–216. 10.1016/S1474-4422(12)70291-0 23332364 PMC3622225

[B20] Jack C. R. Jr. Knopman D. S. Jagust W. J. Shaw L. M. Aisen P. S. Weiner M. W.. (2010). Hypothetical model of dynamic biomarkers of the Alzheimer’s pathological cascade. Lancet Neurol. 9, 119–128. 10.1016/S1474-4422(09)70299-6 20083042 PMC2819840

[B21] Loewenstein D. A. Barker W. W. Chang J. Y. Apicella A. Yoshii F. Kothari P.. (1989). Predominant left hemisphere metabolic dysfunction in dementia. Arch. Neurol. 46, 146–152. 10.1001/archneur.1989.00520380046012 2783845

[B22] Luck T. Pabst A. Rodriguez F. S. Schroeter M. L. Witte V. Hinz A.. (2018). Age-, sex- and education-specific norms for an extended CERAD neuropsychological assessment battery-results from the population-based LIFE-adult-study. Neuropsychology 32, 461–475. 10.1037/neu0000440 29517259

[B23] Marečová P. Zahálková V. (2016). The economic burden of the care and treatment for people with Alzheimer’s disease: the outlook for the Czech Republic. Neurol. Sci. 37, 1917–1922. 10.1007/s10072-016-2679-6 27470305

[B24] Minkova L. Habich A. Peter J. Kaller C. P. Eickhoff S. B. Klöppel S. (2017). Gray matter asymmetries in aging and neurodegeneration: a review and meta-analysis. Hum. Brain Mapp. 38, 5890–5904. 10.1002/hbm.23772 28856766 PMC6866813

[B25] Minoshima S. Frey K. A. Koeppe R. A. Foster N. L. Kuhl D. E. (1995). A diagnostic approach in Alzheimer’s disease using three-dimensional stereotactic surface projections of fluorine-18-FDG PET. J. Nucl. Med. 36, 1238–1248. 7790950

[B26] Morris J. C. Heyman A. Mohs R. C. Hughes J. P. van Belle G. Fillenbaum G.. (1989). The consortium to establish a registry for Alzheimer’s disease (CERAD). Part I. Clinical and neuropsychological assessment of Alzheimer’s disease. Neurology 39, 1159–1165. 10.1212/wnl.39.9.1159 2771064

[B27] Murayama N. Ota K. Kasanuki K. Kondo D. Fujishiro H. Fukase Y.. (2016). Cognitive dysfunction in patients with very mild Alzheimer’s disease and amnestic mild cognitive impairment showing hemispheric asymmetries of hypometabolism on ^18^F-FDG PET. Int. J. Geriatr. Psychiatry 31, 41–48. 10.1002/gps.4287 25820930

[B29] Nobili F. Arbizu J. Bouwman F. Drzezga A. Agosta F. Nestor P.. (2018). European association of nuclear medicine and european academy of neurology recommendations for the use of brain ^18^F-fluorodeoxyglucose positron emission tomography in neurodegenerative cognitive impairment and dementia: Delphi consensus. Eur. J. Neurol. 25, 1201–1217. 10.1111/ene.13728 29932266

[B30] Peña-Longobardo L. M. Oliva-Moreno J. (2015). Caregiver burden in Alzheimer’s disease patients in spain. J. Alzheimers Dis. 43, 1293–1302. 10.3233/JAD-141374 25159671

[B31] Perani D. Schillaci O. Padovani A. Nobili F. M. Iaccarino L. Della Rosa P. A.. (2014). A survey of FDG- and amyloid-PET imaging in dementia and GRADE analysis. Biomed. Res. Int. 2014:785039. 10.1155/2014/785039 24772437 PMC3977528

[B32] Salmon E. Bernard Ir C. Hustinx R. (2015). Pitfalls and limitations of PET/CT in brain imaging. Semin. Nucl. Med. 45, 541–551. 10.1053/j.semnuclmed.2015.03.008 26522395

[B33] Schönknecht O. D. P. Hunt A. Toro P. Guenther T. Henze M. Haberkorn U.. (2011). Bihemispheric cerebral FDG PET correlates of cognitive dysfunction as assessed by the CERAD in Alzheimer’s disease. Clin. EEG Neurosci. 42, 71–76. 10.1177/155005941104200207 21675596

[B34] Shi F. Liu B. Zhou Y. Yu C. Jiang T. (2009). Hippocampal volume and asymmetry in mild cognitive impairment and Alzheimer’s disease: meta-analyses of MRI studies. Hippocampus 19, 1055–1064. 10.1002/hipo.20573 19309039

[B35] Sperling R. A. Aisen P. S. Beckett L. A. Bennett D. A. Craft S. Fagan A. M.. (2011). Toward defining the preclinical stages of Alzheimer’s disease: recommendations from the National institute on aging-Alzheimer’s association workgroups on diagnostic guidelines for Alzheimer’s disease. Alzheimers Dement. 7, 280–292. 10.1016/j.jalz.2011.03.003 21514248 PMC3220946

[B36] Staffaroni A. M. Melrose R. J. Leskin L. P. Riskin-Jones H. Harwood D. Mandelkern M.. (2017). The functional neuroanatomy of verbal memory in Alzheimer’s disease: [^18^F]-fluoro-2-deoxy-D-glucose positron emission tomography (FDG-PET) correlates of recency and recognition memory. J. Clin. Exp. Neuropsychol. 39, 682–693. 10.1080/13803395.2016.1255312 27876444

[B37] Teipel S. J. Willoch F. Ishii K. Bürger K. Drzezga A. Engel R.. (2006). Resting state glucose utilization and the CERAD cognitive battery in patients with Alzheimer’s disease. Neurobiol. Aging 27, 681–690. 10.1016/j.neurobiolaging.2005.03.015 15908048

[B38] Wachinger C. Nho K. Saykin A. J. Reuter M. Rieckmann A. Alzheimer’s Disease Neuroimaging Initiative. (2018). A longitudinal imaging genetics study of neuroanatomical asymmetry in Alzheimer’s disease. Biol. Psychiatry 84, 522–530. 10.1016/j.biopsych.2018.04.017 29885764 PMC6123250

[B39] Wachinger C. Salat D. H. Weiner M. Reuter M. Alzheimer’s Disease Neuroimaging Initiative. (2016). Whole-brain analysis reveals increased neuroanatomical asymmetries in dementia for hippocampus and amygdala. Brain 139, 3253–3266. 10.1093/brain/aww243 27913407 PMC5840883

[B40] Weise C. M. Chen K. Chen Y. Kuang X. Savage C. R. Reiman E. M.. (2018). Left lateralized cerebral glucose metabolism declines in amyloid-β positive persons with mild cognitive impairment. Neuroimage Clin. 20, 286–296. 10.1016/j.nicl.2018.07.016 30101060 PMC6084012

[B41] Welsh K. A. Hoffman J. M. Earl N. L. Hanson M. W. (1994). Neural correlates of dementia: regional brain metabolism (FDG-PET) and the CERAD neuropsychological battery. Arch. Clin. Neuropsychol. 9, 395–409. 14589655

[B42] Wolfsgruber S. Jessen F. Wiese B. Stein J. Bickel H. Mösch E.. (2014). The CERAD neuropsychological assessment battery total score detects and predicts Alzheimer disease dementia with high diagnostic accuracy. Am. J. Geriatr. Psychiatry 22, 1017–1028. 10.1016/j.jagp.2012.08.021 23759289

[B43] Zahn R. Juengling F. Bubrowski P. Jost E. Dykierek P. Talazko J.. (2004). Hemispheric asymmetries of hypometabolism associated with semantic memory impairment in Alzheimer’s disease: a study using positron emission tomography with fluorodeoxyglucose-F^18^. Psychiatry Res. 132, 159–172. 10.1016/j.pscychresns.2004.07.006 15598550

